# Assessment of Patellar Morphology in Trochlear Dysplasia on Computed Tomography Scans

**DOI:** 10.1111/os.12825

**Published:** 2021-01-24

**Authors:** Ming Li, Gang Ji, Liu Fan, Chong‐yi Fan, Wei Lin, Guang‐min Yang, Cong‐lei Dong, Wang Fei

**Affiliations:** ^1^ Department of Orthopaedic Surgery Third Hospital Shijiazhuang China; ^2^ Department of Internal Neurology Second Hospital Hebei Medical University Shijiazhuang China

**Keywords:** Patellar instability, Trochlear dysplasia, Patellar morphology change, CT scan

## Abstract

**Objective:**

To evaluate the patellar morphology of trochlear dysplasia and normal knees in different genders and in different severities of trochlear dysplasia on CT scans.

**Methods:**

A total of 75 patients with trochlear dysplasia (110 knees) treated at the Third Hospital of Hebei Medical University from December 2013 to December 2018 were included in an experimental group, and an age‐matched and sex‐matched cohort of 46 patients with normal trochlear shape (61 knees) were randomly selected into a control group. The experimental group was divided into a female experimental group (Group FE, 47 patients, 72 knees) and a male experimental group (Group ME, 28 patients, 38 knees); the control group was divided into a female control group (Group FC, 31 knees, 24 female patients) and a male control group (Group MC, 30 knees, 22 male patients). Furthermore, according to the severity of trochlear dysplasia, Group FE was divided into a female low‐grade dysplasia group (Group FL, 20 knees) and a female high‐grade dysplasia group (Group FH, 52 knees); Group ME was divided into a male low‐grade dysplasia group (Group ML, 16 knees) and a male high‐grade dysplasia group (Group MH, 22 knees). All participants had undergone CT scans in the supine position; the patellar width and thickness, the lateral patellar facet angle, the Wiberg angle, and the Wiberg index were measured and compared.

**Results:**

In trochlear dysplasia knees, the mean patellar width and thickness and the lateral patellar facet angle were significantly smaller; the mean Wiberg index was significantly larger than in normal knees, regardless of gender (*P* < 0.05); and there was no statistically significant difference in the mean Wiberg angle (*P* > 0.05). In the female groups, the mean patellar width and thickness and the Wiberg angle were significantly smaller; the mean lateral patellar facet angle was significantly larger than those in the male groups (*P* < 0.05); and there was no significant difference in the mean Wiberg index (*P* > 0.05). In the low‐grade dysplasia group, the mean Wiberg index was smaller than that in the high‐grade dysplasia group (*P* < 0.05), regardless of gender; however, there was no significant difference in the mean patellar width and thickness, the lateral patellar facet angle, and the Wiberg angle in low‐grade and high‐grade dysplasia (*P* > 0.05).

**Conclusion:**

On CT scans, the patella in trochlear dysplasia had a smaller width, a thinner thickness, a lengthened lateral facet, and a more flattened articular facet. In addition, the patellar articular facet was more prominent in female patients. With the severity of trochlear dysplasia increased, the lateral patellar facet became longer. In addition, the abnormal stress distribution on the patella influenced the patellar morphology in trochlear dysaplasia.

## Graphical abstract

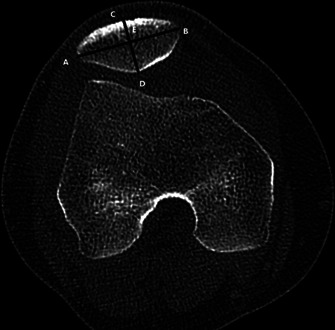

The author found the patella in trochlear dysplasia have a smaller width, a thinner thickness, a lengthened lateral facet, and a more flattened articular facet. In addition, the patellar articular facet was more prominent in female patients. With the severity of trochlear dysplasia increased, the lateral patellar facet became longer.

## Introduction

Patellar instability is a common knee disease in children aged 10 to 17 years, and the incidence of primary patellar dislocation is approximately 21 per 100,000 individuals^1^. The function and the stability of the patellofemoral joint are normally maintained by a complex interaction among the active stabilizers, the passive stabilizers, and the osseous and cartilage morphology^2^, ^3^. Issues with any of the three factors can lead to patellar instability^4^.

Trochlear dysplasia, which belongs to the osseous morphology abnormal, is defined by a decreased trochlear depth and flattened lateral or medial condylar height^5^, and exists in 96% patients who have patellar instability^6^. In 1964, Brattström^7^ reported the abnormal shape of the trochlear groove in patients with patellar instability, and Malghem and Maldague^8^ later quantified trochlear depth on lateral radiographs. Since then, doctors have begun to attach importance to trochlea dysplasia and it is currently universally recognized as one of the most important predisposing factors for patellar instability^6^, ^9^, ^10^ and failed surgical stabilization^11^.

Over the past few decades, trochlear morphology in trochlear dysplasia has been thoroughly investigated^5^, ^6^. In 2018, Dejour and Coultre classified the trochlear morphology in trochlear dysplasia: type A trochlea had a fairly shallow surface; type B trochlea were flat or convex; type C trochlea had a convex lateral facet and a hypoplastic medial facet; and type D had asymmetry of the trochlea facets, like type C, and a cliff pattern^12^.

As for the patella, most studies concentrate on its position relative to the trochlear groove, such as the patellar height, the patellar tilt angle, and the congruence angle. Studies focusing on changes in the patellar morphology in trochlear dysplasia are rare^13^, ^14^, ^15^, ^16^, ^17^.

The patella is shaped like an upside‐down triangle, and its anatomy reveals a median crest traversing the articular part of the patella, defining a medial and a lateral facet. In 1941, Wiberg^18^ classified patellar morphology as follows: type I patella had two concave facets of approximately equal size; type II patella had two concave facets where the medial facet was smaller than the lateral facet; and type III patella had a convex medial facet and a concave lateral facet.

Sandro *et al*.^13^ and Yılmaz *et al*.^14^ reported that patients with trochlear dysplasia had a smaller transverse and vertical patellar size, and the length of the medial facet was shortened in comparison with normal knees. Askenberger *et al*.^15^ reported that in skeletally immature children with a primary patellar dislocation, the incidence of Wiberg type III patella was significantly higher than that in patients without patellar dislocation. Servien *et al*.^16^ also found that a patella with a hypoplasic medial border, a Wiberg type III patella, or a short patellar apex was most likely to present in the patellar dislocation patients. Pfirrmann *et al*.^17^ further showed that the width of the medial facet was 12% of that of the lateral facet in patients with trochlear dysplasia, but in normal knees, the mean was 57%.

However, the previous studies were limited by small sample size. Studies have reported that the risk of patellar dislocation among girls is three times higher than that for boys and that patellar morphology is significantly different between the genders^1^, ^19^. However, there is no study that focuses on this difference, which might lead to deviations. In addition, Lippacher *et al*.^20^ put forward a two‐grade classification for trochlear dysplasia: type A dysplasia was low‐grade dysplasia, and types B, C, and D dysplasia were high‐grade dysplasia. Patellar morphology may change with different severities of trochlear dysplasia, which has been ignored in previous studies.

Consequently, we evaluated the patellar morphology change in trochlear dysplasia and normal knees on CT scans for a large sample. All data was separately analyzed based on different genders and different severity of trochlear dysplasia.

Furthermore, the etiology of the trochlear as well as the changes in patellar morphology in trochlear dysplasia remain unclear. Studies have reported that trochlear dysplasia is determined in utero, that the trochlear morphology would not change during growth^21^, ^22^ that genetic predisposition is a primary cause of trochlear dysplasia. In contrast, other studies show that the trochlear morphlogy is changed after surgical correction in skeletally immature patients; the acquired factors necessarily influence the trochlear dysplasia^23^, ^24^.

Therefore, the purpose of the present study was to: (i) evaluate the patellar morphology of trochlear dysplasia and normal knees in different genders; (ii) identify the changes in patellar morphology with different severities of trochlear dysplasia; and (iii) explore the etiology of trochlear dysplasia.

We hypothesized that on CT scans, the patella in trochlear dysplasia would obviously change; it might have a smaller width, a thinner thickness, a lengthened lateral facet, and a more flattened patellar articular facet. The patellar articular facet was more flattened in female patients. In addition, the patella morphology might have different representations in various severities of trochlear dysplasia. The abnormal stress distribution on the patella necessarily influenced patellar morphology in trochlear dysaplasia.

## Materials and Methods

### *Patients*

The present study was approved by the Academic Ethics Committee of the Third Hospital of Hebei Medical University and all patients provided informed consent.

In the present study, 75 patients (110 knees, mean age, 32.54 years) were included in the experimental group. The inclusion criteria were as follows: (i) patients treated at the Third Hospital of Hebei Medical University from December 2013 to December 2018; (ii) patients aged from 18 to 45 years; (iii) patients that had experienced more than two episodes of dislocation or one episode of dislocation plus multiple episodes of instability, or patellar instability following the initial dislocation had persisted for more than 3 months; (iv) patients that had undergone CT examination of the knee joint in the supine position; and (v) patients with trochlear dysplasia that was diagnosed according to Dejour and Coultre on CT scan^12^.

Our exclusion criteria were: (i) previous knee surgery; (ii) acute patellar dislocation; (iii) rheumatoid arthritis^25^; and (iv) patellofemoral arthritis greater than grade II, with the patellofemoral joint surface having bone contact (Iwano classification)^26^.

A total of 14 patients were excluded from the experimental group: 1 patient had previous knee surgery; 8 patients had an acute dislocation; and 5 patients had patellofemoral arthritis greater than grade II (Iwano classification).

The control group which was matched with the experimental group according to sex and age included 46 patients with normal trochlear shape (61 knees, mean age 36.54 years). The patients underwent knee CT examinations due to anterior cruciate ligament and posterior cruciate ligament rupture and were consecutively collected during the same period. According to gender, the experimental group was divided into Group FE (72 knees, 47 female patients) and Group ME (38 knees, 28 male patients), and the control group was divided into Group FC (31 knees, 24 female patients) and Group MC (30 knees, 22 male patients).

**Fig. 1:**
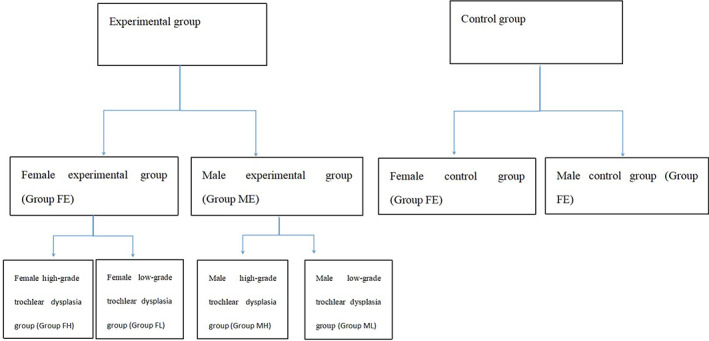
Division of experimental and control groups.

According to the severity of trochlear dysplasia^20^, Group FE was divided into Group FL (22 low‐grade dysplasia knees) and Group FH (50 high‐grade dysplasia knees); Group ME was divided into Group ML (16 low‐grade dysplasia knees) and Group MH (22 high‐grade dysplasia knees) (Fig. 1) .

The diagnosis was confirmed by two authors (Li and Ji) based on the axial CT scans. The demographics of the patients are summarized in Tables 1 and 2.

**TABLE 1:** The demographics of the female groups

Groups	Patients number	Sample size	Mean age (years)	Side (left/right)	BMI (kg/m^2^)	Low‐/high‐ grade dysplasia
Group FE	47	72	29.7 ± 7.8	32/40	22.49 ± 3.21	20/52
Group FC	24	31	35.2 ± 6.4	13/18	22.43 ± 2.97	—*
*P* value	—	—	—	—	0.942	—

Group FE, female experimental group; Group FC, female control group.

* There was no low‐ or high‐ grade dysplasia in the Group FC, patients in the group had normal trochlear shape.

**TABLE 2:** The demographics of the male groups

Groups	Patients number	Sample size	Mean age (years)	Side (left/right)	BMI (kg/m^2^)	Low‐/high‐ grade dysplasia
Group ME	28	38	27.6 ± 8.0	22/16	23.15 ± 2.65	16/22
Group MC	22	30	31.2 ± 6.1	13/8	24.11 ± 2.17	—
*P* value	—	—	—	—	0.292	—*

Group ME, male experimental group; Group MC, male control group.

* There was no low‐ or high‐ grade dysplasia in the Group MC, patients in the group had normal trochlear shape.

### *CT* *Protocols*

All patients underwent CT examination in the supine position, with 20° of knee flexion. The limbs were fixed by equipment to minimize motion. All examinations were performed with the same CT scanner (SOMATOM Sensation 16; Siemens Medical Solutions, Erlangen, Germany). The CT scanning parameters included a tube voltage of 120 kV, 100 effective mAs, 1‐mm slice thickness, a gantry rotation time of 1 s, and a matrix size of 512 × 512. All measurements were performed using RadiAnt DICOM software (Medical, Poznan, Poland).

### *Assessments*

Trochlear morphology was classified according to Dejour and Coultre^12^; the patellar width and thickness, the lateral patellar facet angle, the Wiberg angle, and the Wiberg index were measured on the axial CT scans. Our measurement methods had an accuracy of 0.01 mm and 0.1°. The two authors were blinded to the characteristics of the patients and obtained all measurements independently. Intraclass correlation coefficient values (ICC) were calculated to test intraobserver and interobserver reliability.

#### *Patellar Width*

**Fig. 2:**
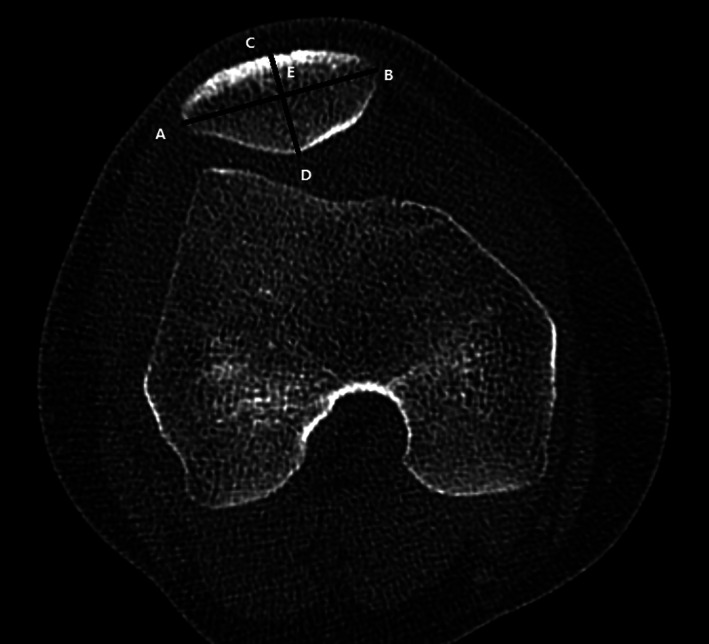
The patellar width (AB) is the length between the medial (A) and lateral edge (B) of the patella. The patellar thickness (CD) is the length between the patellar front polar (C) and back polar (D). The Wiberg index is defined as the ratio of the transverse length of the lateral patellar facet (AE) to the patellar width (AB).

The patellar width (AB) is defined as the length between the medial (A) and lateral edge (B) of the patella in the slide with the widest patellar diameter (Fig. 2). The patellar width reflects the transverse length of the patella^27^.

#### *Patellar Thickness*

The patellar thickness (CD) is defined as the length between the patellar front polar (C) and back polar (D) (Fig. 2). The patellar thickness reflects the vertical length of the patella on the axial CT scans.

#### *Lateral Patellar Facet Angle*

**Fig. 3:**
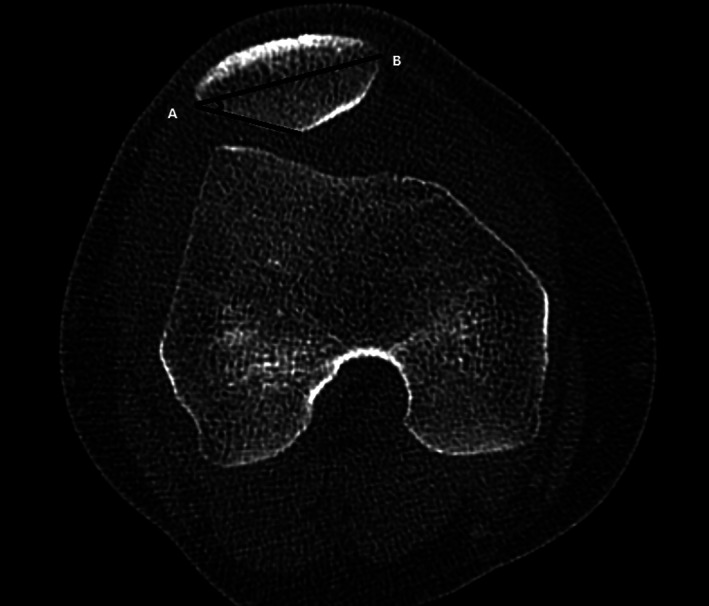
The lateral patellar facet angle is the angle formed by the patellar transverse axis (AB) and the lateral patellar facet tangent.

The lateral patellar facet angle is defined as the angle formed by the patellar transverse axis (AB) and the lateral patellar facet tangent (Fig. 3). A higher angle means the patellar articular surface is more prominent.

#### *Wiberg Angle*

**Fig. 4:**
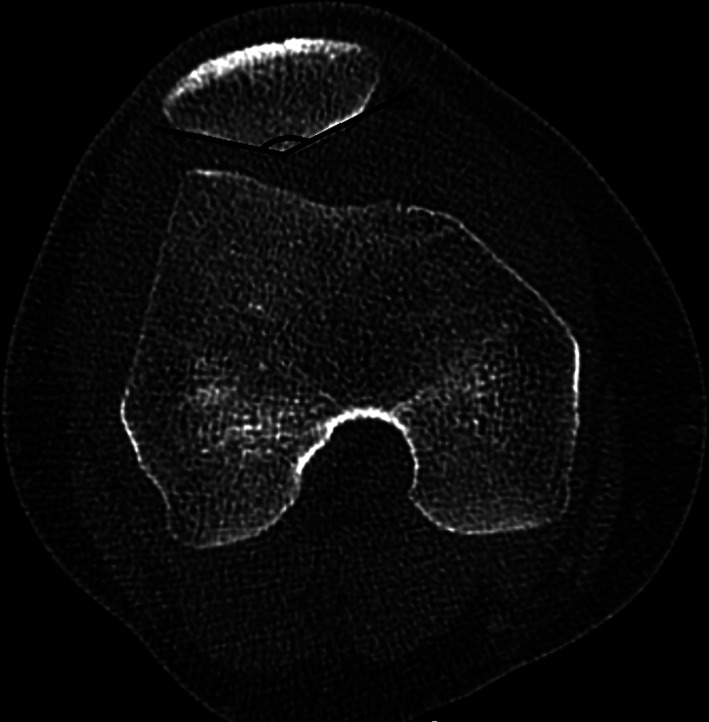
The Wiberg angle is the angle formed by the medial and the lateral patellar facet tangent.

The Wiberg angle is defined as the angle formed by the medial and the lateral patellar facet tangent^13^. (Fig. 4). A higher angle means the patellar articular surface is more flattened.

#### *Wiberg Index*

The Wiberg index is defined as the ratio of the transverse length of the lateral patellar facet (AE) to the patellar width (AB)^13^ (Fig. 2). A higher Wiberg index means that hypoplasia in the medial patellar facet and hyperplasia in the lateral patellar facet are more obvious.

### *Statistical Analysis*

The SPSS v13.0 statistical software was used to analyze our data. The Kolmogorov–Smirnov normality test was performed to test whether the data fit a Gaussian distribution. The patellar width, the Wiberg index, and the Wiberg angle of Group FE did not pass the Kolmogorov–Smirnov text, so the data were analyzed using the Mann–Whitney test; the other data were analyzed using Student's paired *t*‐test. *P*‐values less than 0.05 were defined as significantly different.

## Results

In this study, all data were expressed as mean ± standard deviation. There were no significant differences in body mass index between Group FE and Group FC (*P* = 0.942), and Group ME and Group MC (*P* = 0.292, Tables 1 and 2).

### *Intrarater and Interrater Variability*

The intrarater reliability was excellent for all the measurements (*ICC* = 0.988; 95%*CI*, 0.985–0.991) and the interrater reliability was high (*ICC* = 0.992; 95% *CI* 0.990–0.994).

### *Smaller Width, Thinner Thickness, Lengthened Lateral Facet, and a More Flattened Articular Facet in Trochlear Dysplasia*

In Group FE, the mean patellar width and thickness and the lateral patellar facet angle were 38.52 ± 2.48 mm, 18.86 ± 1.45 mm, and 23.5° ± 2.9°; the mean Wiberg angle and Wiberg index were 130.3° ± 6.8° and 0.62 ± 0.05. In Group FC, the mean patellar width and thickness and the lateral patellar facet angle were 39.72 ± 2.15 mm, 19.47 ± 1.23 mm, and 26.0° ± 2.6°; the mean Wiberg angle and Wiberg index were 129.2° ± 4.5° and 0.56 ± 0.03.

In Group ME, the mean patellar width and thickness and the lateral patellar facet angle were 45.22 ± 2.43 mm, 21.22 ± 1.48 mm, and 21.8° ± 3.5°; the mean Wiberg angle and Wiberg index were 137.2° ± 5.7° and 0.61 ± 0.05. In Group MC, the mean patellar width and thickness and the lateral patellar facet angle were 46.46 ± 2.33 mm, 21.97 ± 1.46 mm, and 24.1° ± 2.9°; the mean Wiberg angle and Wiberg index were 135.0° ± 5.2° and 0.56 ± 0.03.

We separately compared Group FE and Group FC, and Group ME and Group MC. In trochlear dysplasia knees, the mean patellar width and thickness and the lateral patellar facet angle were significantly smaller (*P* < 0.05); the mean Wiberg index was larger (*P* < 0.05) than in normal knees, regardless of gender.

There was no significant difference in the mean Wiberg angle between the experimental and control groups (*P* > 0.05) (Tables 3 and 4).

**TABLE 3:** Comparison of patellar morphology among trochlear dysplasia knees and normal knees in the female groups

Groups	Patellar width (mm)	Patellar thickness (mm)	Patellar lateral facet angle (°)	Wiberg angle (°)	Wiberg index
Group FE	**38.52 ± 2.48**	**18.86 ± 1.45**	**23.5 ± 2.9**	130.3 ± 6.8	**0.62 ± 0.05**
Group FC	**39.72 ± 2.15**	**19.47 ± 1.23**	**26.0 ± 2.6**	129.2 ± 4.5	**0.56 ± 0.03**
*P* value	**0.022**	**0.042**	**0.000**	0.459	**0.000**

Group FE, female experimental group; Group FC, female control group. Significant differences were indicated in bold.

**TABLE 4:** Comparison of patellar morphology among trochlear dysplasia knees and normal knees in the male groups

Groups	Patellar width (mm)	Patellar thickness (mm)	Patellar lateral facet angle (°)	Wiberg angle (°)	Wiberg index
Group ME	**45.22 ± 2.43**	**21.22 ± 1.48**	**21.8 ± 3.5**	137.2 ± 5.7	**0.61 ± 0.05**
Group MC	**46.46 ± 2.33**	**21.97 ± 1.46**	**24.1 ± 2.9**	135.0 ± 5.2	**0.56 ± 0.03**
*P* value	**0.047**	**0.049**	**0.006**	0.136	**0.000**

Group ME, male experimental group; Group MC, male control group. Significant differences were indicated in bold.

### *Patellar Articular Facet is More Prominent in Female Patients*

We separately compared Group FE and Group ME, and Group FC and Group MC. In the female groups, the patellar width and thickness and the Wiberg angle were significantly smaller (*P* < 0.05); the lateral patellar angle was bigger than those in the male groups (*P* < 0.05); this was regardless of trochlear dysplasia or normal knees. There was no significant difference in the Wiberg index (*P* > 0.05). (Tables 5 and 6).

**TABLE 5:** Comparison of patellar morphology between genders in the experimental groups

Groups	Patellar width (mm)	Patellar thickness (mm)	Lateral patellar facet angle (°)	Wiberg angle (°)	Wiberg index
Group FE	**38.52 ± 2.48**	**18.86 ± 1.45**	**23.5 ± 2.9**	**130.3 ± 6.8**	0.62 ± 0.05
Group ME	**45.22 ± 2.43**	**21.22 ± 1.48**	**21.8 ± 3.5**	**137.2 ± 5.7**	0.61 ± 0.05
*P* value	**0.000**	**0.000**	**0.007**	**0.000**	0.538

Group FE, female experimental group; Group ME, male experimental group. Significant differences were indicated in bold.

**TABLE 6:** Comparison of patellar morphology between genders in the control groups

Groups	Patellar width (mm)	Patellar thickness (mm)	Lateral patellar facet angle (°)	Wiberg angle (°)	Wiberg index
Group FC	**39.72 ± 2.15**	**19.47 ± 1.23**	**26.0 ± 2.6**	**129.2 ± 4.5**	0.56 ± 0.03
Group MC	**46.46 ± 2.33**	**21.97 ± 1.46**	**24.1 ± 2.9**	**135.0 ± 5.2**	0.56 ± 0.03
*P* value	**0.000**	**0.000**	**0.014**	**0.000**	0.902

Group FC, female control group; Group MC, male control group. Significant differences were indicated in bold.

### *Lateral Patellar Facet is Longer in* *High‐Grade* *Dysplasia*

In Group FL, the mean patellar width and thickness and the lateral patellar facet angle were 38.70 ± 2.23 mm, 18.88 ± 1.12 mm, and 24.2° ± 2.9°; the mean Wiberg angle and Wiberg index were 128.2° ± 6.8° and 0.59 ± 0.04. In Group FH, the mean patellar width and thickness and the lateral patellar facet angle were 38.41 ± 2.56 mm, 18.87 ± 1.57 mm, and 23.3° ± 3.0°; the mean Wiberg angle and Wiberg index were 130.6° ± 6.7° and 0.62 ± 0.05.

In Group ML, the mean patellar width and thickness and the lateral patellar facet angle were 45.36 ± 2.37 mm, 21.66 ± 1.68 mm, and 22.9° ± 3.6°; the mean Wiberg angle and Wiberg index were 136.8° ± 4.9° and 0.59 ± 0.04. In Group MH, the mean patellar width and thickness and the lateral patellar facet angle were 45.12 ± 2.52 mm, 20.91 ± 1.28 mm, and 21.0° ± 3.3°; the mean Wiberg angle and Wiberg index were 137.5° ± 6.3° and 0.63 ± 0.04.

We separately compared Group FL and Group FH, and Group ML and Group MH. In low‐grade dysplasia, the Wiberg index was significantly smaller than that in high‐grade dysplasia (*P* < 0.05), regardless of gender.

There was no significant difference in the patellar width and thickness and the lateral patellar facet angle, and the Wiberg angle in low‐grade and high‐grade dysplasia (*P* > 0.05) (Tables 7 and 8).

**TABLE 7:** Comparison of patellar morphology among low‐ and high‐grade dysplasia groups in the female groups

Groups	Patellar width (mm)	Patellar thickness (mm)	Lateral Patellar facet angle (°)	Wiberg angle (°)	Wiberg index
Group FL	38.70 ± 2.23	18.88 ± 1.12	24.2 ± 2.9	128.2 ± 6.8	**0.59 ± 0.04**
Group FH	38.41 ± 2.56	18.87 ± 1.57	23.3 ± 3.0	130.6 ± 6.7	**0.62 ± 0.05**
*P* value	0.721	0.937	0.245	0.201	**0.004**

Group FL, female low‐ grade dysplasia group; Group FH, female high‐ grade dysplasia group. Significant differences were indicated in bold.

**TABLE 8:** Comparison of patellar morphology among low‐ and high‐grade dysplasia groups in the male groups

Groups	Patellar width (mm)	Patellar thickness (mm)	Lateral patellar facet angle (°)	Wiberg angle (°)	Wiberg index
Group ML	45.36 ± 2.37	21.66 ± 1.68	22.9 ± 3.6	136.8 ± 4.9	**0.59 ± 0.04**
Group MH	45.12 ± 2.52	20.91 ± 1.28	21.0 ± 3.3	137.5 ± 6.3	**0.63 ± 0.04**
*P* value	0.772	0.133	0.644	0.738	**0.006**

Group ML, male low‐ grade dysplasia group; Group MH, male high‐grade dysplasia group. Significant differences were indicated in bold.

## Discussion

The most important finding of this study is that the patella in trochlear dysplasia had a smaller width, a thinner thickness, a lengthened lateral facet, and a more flattened patellar articular facet than in normal knees, regardless of gender; and the patella in female patients had a more prominent articular facet than that in male patients; in addition, with the severity of trochlear dysplasia increased, the lateral patellar facet became longer. As far as we know, this is the first study to evaluate the patellar morphology in trochlear dysplasia and normal knees on CT scans and to separately analyze the data according to gender and severity of trochlear dysplasia.

### *Etiology of Trochlear Dysplasia*

Up to now, controversy has remained with regard to the etiology of trochlear dysplasia. Studies have reported that trochlear dysplasia is determined early in utero, and the abnormal shape is maintained during growth^21^, ^22^. Øye *et al*.^28^ found that children with a breech presentation had a high risk of congenital trochlear dysplasia. These studies supported that genetic predisposition was a primary cause of trochlear dysplasia.

However, Benoit and Fu *et al*.^23^, ^24^ showed that surgical correction could remodel the dysplastic trochlea in skeletally immature patients. In addition, experimental studies have reported that the trochlear morphology is obviously changed in growing rabbits after patellar malposition^29^, ^30^. Therefore, acquired factors also necessarily affect the trochlear dysplasia.

### *Abnormal Stress Distribution Influences Patellar Morphology in Trochlear Dysplasia*

Van Haver *et al*.^10^ found that the patella in trochlear dysplasia showed lateral tilt and lateral translation; the articular surface had an increased contact pressure and a decreased contact area. In our opinion, the lateralized patella led the stress between the patellofemoral joint, which was excessively concentrated and shifted laterally. The excessive mechanical stress on the lateral trochlear and lateral patellar facet caused hyperplasia; the center and the medial trochlear surface, as well as the medial patellar facet and the patellar crest, represented hypoplasia because of the insufficient stress. The abnormal stress distribution, which was an acquired factor, played an important role in the changes in the morphology of the trochlea and patella in trochlear dysplasia.

In the present study, we found that the medial patellar facet was shorter and the lateral patellar facet was longer in trochlear dysplasia; in other words, there was hypoplasia in the medial facet and hyperplasia in the lateral facet. Meanwhile, the thinned patellar thickness and the flattened patellar articular surface signified that the patellar crest also had hypoplasia. The changes in the patellar morphology confirmed our opinion. We further found that as the severity of trochlear dysplasia worsened, the proportion of the transverse length of the lateral patellar facet to the patellar width was increased. Studies have reported that the lateral tilt and lateral translation of the patella were more evident in trochlear dysplasia with a pronounced trochlear bump^10^, which was represented in high‐grade dysplasia. Therefore, the lateral patellar facet was under more stress and the hyperplasia was more serious in high‐grade dysplasia.

In trochlear dysplasia, the patellar width was shortened, which means that although hypoplasia and hyperplasia both existed in the patella, the patella presented a trend of dysplasia overall. Therefore, patients with trochlear dysplasia were accompanied by patellar dysplasia.

Between genders, we found that the patellar width and thickness were significantly smaller in the female groups than in the male groups, which was consistent with the previous studies^19^. Interestingly, studies have reported that the risk for patellar dislocation among girls is three times higher than that for boys^1^, and we hypothesized that the patellar articular facet was more flattened in female patients. However, in the present study, the Wiberg angle was significantly smaller and the lateral patellar angle was bigger in the female groups, which meant that the patella in women had a more prominent articular facet, which was contrary to our hypothesis. This may be because women have a larger Q angle which results in a greater force that pulls the patella laterally^31^. In order to obtain a more stable patellofemoral joint, women are born with a more prominent patella articular surface. Further studies should concentrate on this difference of the patellar morphology between genders.

In addition, the Wiberg angle was bigger in trochlear dysplasia but not statistically significantly. We measured another parameter, the lateral patellar facet angle, which was formed by the patellar transverse axis and the lateral patellar facet tangent. The angle was more sensitive and could directly inflect the prominence of the patellar articular facet.

### *Limitations*

There were obvious limitations to this study. The sample size of the male groups was small and the present study was a single‐center retrospective study, which could lead to deviations; in addition, we used the transverse sections only, which might not reflect all the morphological changes of the patella. Consequently, sagittal sections should also be considered in the assessment.

### *Conclusion*

On CT scans, the patella in trochlear dysplasia had a smaller width, a thinner thickness, a lengthened lateral facet, and a more flattened articular facet. In addition, the patellar articular facet was more prominent in female patients. With the severity of trochlear dysplasia increased, the lateral patellar facet became longer.
